# Concurrent Viewing of H&E and Multiplex Immunohistochemistry in Clinical Specimens

**DOI:** 10.3390/diagnostics15020164

**Published:** 2025-01-13

**Authors:** Larry E. Morrison, Tania M. Larrinaga, Brian D. Kelly, Mark R. Lefever, Rachel C. Beck, Daniel R. Bauer

**Affiliations:** Roche Diagnostics Solutions (Ventana Medical Systems, Inc.), 1910 E. Innovation Park Dr., Tucson, AZ 85755, USA; lemorrison73@gmail.com (L.E.M.);

**Keywords:** immunohistochemistry (IHC), multiplexing, hematoxylin and eosin (H&E), chromogens, brightfield microscopy, multispectral imaging

## Abstract

**Background/Objectives:** Performing hematoxylin and eosin (H&E) staining and immunohistochemistry (IHC) on the same specimen slide provides advantages that include specimen conservation and the ability to combine the H&E context with biomarker expression at the individual cell level. We previously used invisible deposited chromogens and dual-camera imaging, including monochrome and color cameras, to implement simultaneous H&E and IHC. Using this approach, conventional H&E staining could be simultaneously viewed in color on a computer monitor alongside a monochrome video of the invisible IHC staining, while manually scanning the specimen. **Methods:** We have now simplified the microscope system to a single camera and increased the IHC multiplexing to four biomarkers using translational assays. The color camera used in this approach also enabled multispectral imaging, similar to monochrome cameras. **Results:** Application is made to several clinically relevant specimens, including breast cancer (HER2, ER, and PR), prostate cancer (PSMA, P504S, basal cell, and CD8), Hodgkin’s lymphoma (CD15 and CD30), and melanoma (LAG3). Additionally, invisible chromogenic IHC was combined with conventional DAB IHC to present a multiplex IHC assay with unobscured DAB staining, suitable for visual interrogation. **Conclusions:** Simultaneous staining and detection, as described here, provides the pathologist a means to evaluate complex multiplexed assays, while seated at the microscope, with the added multispectral imaging capability to support digital pathology and artificial intelligence workflows of the future.

## 1. Introduction

Hematoxylin and eosin (H&E) staining is essential for the evaluation of clinical tissue specimens, with most cancer diagnoses of solid tumors being based on this stain for over a century [1]. Immunohistochemistry (IHC) adds valuable biomarker information for tumor classification, prognosis, and therapeutic decisions [2]. Typically, the biomarker expression and H&E context are obtained by H&E staining one specimen section and performing IHC on serial sections at a minimum of 4 µm apart. However, due to the biological complexity of tissue, the correspondence between individual cells and tissue features on serial sections is not exact, as the same cells are not present on the serial sections and the tissue features change as the serial sections cut through successive regions of tumor. Combining H&E and IHC on the same specimen slide conserves the specimen and places the biomarker expression within the morphological and cellular context provided by H&E to aid current pathologist workflow. Although not used for diagnosis, combining H&E with IHC on the same slide has been reported by performing conventional chromogenic IHC [3] or immunofluorescence [4] (IF), followed by H&E staining. In the former approach, conventional visible deposited chromogens were utilized and H&E and IHC stains were viewed simultaneously using brightfield microscopy. In the latter IF approach, tissue was imaged after the IF staining and again after performing H&E, and the information was then combined and viewed digitally. This is because the strong fluorescence of eosin interferes with IF evaluation. While both methods provided an exact correspondence between the biomarker expression and H&E context, both approaches have drawbacks. Conventional chromogenic IHC employs deposited chromogens absorbing within the visible spectrum, and H&E stains absorb broadly across the visible spectrum. Therefore, the H&E absorbance may obscure the visualization of the IHC stain, and vice versa. In the latter approach, imaging the specimen following IF and a second time after H&E staining is time-consuming and requires the alignment of the IF and H&E images. In addition, in clinical practice, using a microscope, the pathologist cannot view both stains simultaneously or in rapid succession on the actual specimen.

We previously reported the combination of IHC with H&E, but used covalently deposited chromogens (CDCs) that absorb in the invisible portions of the spectrum, the ultraviolet (UV), or near infrared (NIR), to minimize interference between the IHC stain absorbance and that of the H&E stain [5]. Using a dual-camera system, the pathologist was able to view the visible H&E stain through the microscope oculars while a monochrome camera presented live images of the UV- or NIR-absorbing CDCs on the computer monitor beside live color images of the H&E stain. The dual-camera system combines bands of invisible light and visible white light that pass through the specimen, transmitting the visible light to a color camera, and reflecting invisible light to the monochrome camera. When viewing a two-color (duplex) IHC, the two invisible CDCs were alternately viewed on the monochrome video by switching between two different bands of invisible light. This approach allowed the pathologist to view both the H&E and IHC stains simultaneously, while sitting at the microscope with a manual translation of the specimen stage.

By introducing additional invisible CDCs with absorbance maxima strategically placed throughout the UV and NIR spectrum, we now report combining conventional H&E staining with up to four multiplexed biomarkers (4-plex IHC), providing the greater conservation of specimens and analysis of more complex cellular interactions and phenotypes. Moreover, we present a simplified microscope system requiring only a single color camera to sequentially view the H&E and the invisible chromogenic stains. Like the dual-camera system, the single-camera approach supports interactive specimen evaluation by the pathologist while seated at the microscope, as well as multispectral imaging and digital processing. This capability could give the pathologist an unparalleled ability to understand the morphological and protein drivers of the tumor, by allowing them to hone in on specific morphologically relevant regions and identify the biomarker status within those important regions.

## 2. Material and Methods

### 2.1. Chromogens and IHC Reagents

Methods for synthesizing tyramide derivatives of CDCs have been described [6]. Syntheses of tyramide-derivatized dibenzocyclooctyne (DBCO) and azide-modified dyes used in DBCO chemistry to form CDCs have also been described, including the azide of the novel chloro-sCy7 derivative ir870 [5]. Other CDCs included in this work were tyramide-derivatized 7-amino-4-methylcoumarin-3-acetate (AMC), and azide derivatives of 7-(methylamino)coumarin-3-acetate (NMC) and sulfo-Cyanine 7 (sCy7). AMC and sCy7 are also available from several commercial sources.

Chromogens were stored in concentrated stock solutions of DMSO and diluted for storage in DISCOVERY Ultra automated stainer dispensers (Ventana Medical Systems, Inc. (VMSI), Tucson, AZ, USA). The conventional chromogen 3,3′-Diaminobenzidine (DAB) was part of the ultraView Universal DAB Detection Kit (Cat. No. 760–500; VMSI).

The primary antibodies anti-human epidermal growth factor receptor 2 (ERBB2, HER2 or HER2/neu; 4B5, cat no. 790-2991), anti-CD15 (MMA, cat no. 760-2504), anti-CD30 (Ber-H2, cat no. 790-4858), anti-estrogen receptor (ER; SP1, cat no. 790-4325), anti-progesterone receptor (PR; 1E2, cat no. 790-4296), anti-programmed death ligand 1 (PD-L1; SP263, cat no. 740-4907), anti-α-methylacyl CoA racemase (AMACR or P504S; SP116, cat no. 08035130001), anti-CD8 (SP57, cat no. 790–4460), anti-basal cell (cocktail of antibodies to p63 and keratin; 34ßE12+p63, cat no. 790–4536), and prostate-specific membrane antigen (PSMA; EP192) Rabbit Monoclonal Primary Antibody (cat no. 760-6076) were obtained from VMSI. Anti-lymphocyte-activation gene 3 (LAG3) primary antibody (17B4) is available from several commercial sources. VMSI enzyme–antibody conjugates used with CDC detection were OmniMap anti-Ms HRP (RUO), DISCOVERY (cat no. 760-4310), and OmniMap anti-Rb HRP (RUO), DISCOVERY (cat no. 760-4311). HRP multimer detection used DISCOVERY anti-HQ HRP (cat no. 760-4820), DISCOVERY anti-Ms HQ (RUO) (cat no. 760-4814), and DISCOVERY anti-Rb HQ (RUO) (cat no. 760-4815).

### 2.2. Specimens

Formalin-fixed paraffin-embedded (FFPE) slide-mounted sections from normal (relative to cancer) tonsil, breast cancer, melanoma, prostate cancer, and Hodgkin’s lymphoma (nodular sclerosis subtype) were prepared from tissue blocks obtained from the VMSI specimen bank. All specimens were anonymized and consented to meet regulatory requirements.

### 2.3. IHC and Conventional Staining

Fully automated single and multiplex IHC were performed on a DISCOVERY Ultra (VMSI), followed by manual H&E staining. Details of the automated IHC and manual H&E-staining procedures are provided in the Supplementary Methods. DAB IHC was performed using the ultraView Universal DAB Detection Kit (Cat. No. 760–500) with hematoxylin counterstaining (Hematoxylin II, Cat. No. 790-2208/05277965001) and Bluing Reagent (Cat. No. 760-2037), following the manufacturer’s instructions (VMSI). When using DAB with CDCs in multiplex IHC, the CDC IHC was typically performed first. For manual H&E staining, incubation times in hematoxylin and/or eosin were varied to account for reduced or increased staining of tissues after IHC. For example, hematoxylin staining was reduced from 1 min to 10 s and eosin staining was increased from 1 min to 2 min for some prostate and breast specimens.

### 2.4. Microscope Systems

**Figure 1:**
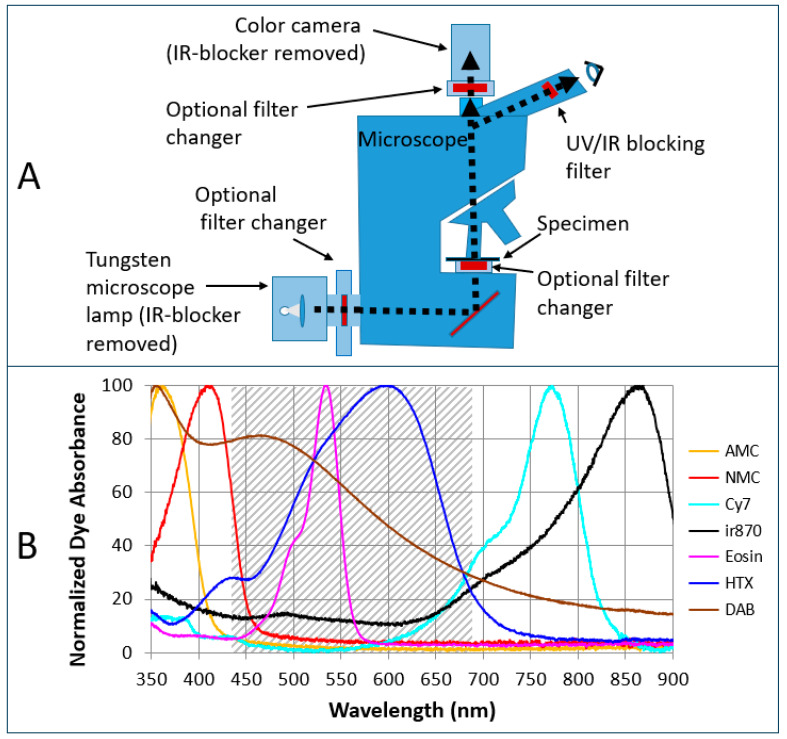
(**A**) Schematic of single-camera microscope system configurations and (**B**) spectra of deposited dye absorbance (amplitude normalized). Shading indicates the spectral region in which the photopic visual response (relative luminosity factors) is >1% of the maximum near 554 nm [7]. HTX = hematoxylin; AMC = tyramide-derivatized 7-amino-4-methylcoumarin-3-acetate; NMC = azide of 7-(methylamino)coumarin-3-acetate; sCy7 or Cy7 = azide of sulfo-Cyanine 7; ir870 = azide of novel chloro-sCy7 derivative; HTX = hematoxylin; DAB = 3,3′-diaminobenzidine.

The dual-camera microscope system that permits simultaneous viewing of color video of H&E stain and monochrome video of non-visible IHC chromogens has been described in detail [5]. The simpler single-camera system that permits sequential viewing of color H&E and invisible IHC videos can be implemented in several ways, depending upon where illumination wavelength selection occurs, as depicted in Figure 1A. The single-camera system included an Olympus BX-63 microscope (Olympus, Waltham, NJ, USA) fitted with Olympus UPlanSApo 20× (NA 0.75) and 10× (NA 0.40) air objectives, a Kiralux, 5.0 Mpixel color camera (model CS505CU; Thorlabs, Newton, NJ, USA), sources of visible white light and invisible light, and a means to select different wavelengths of light. Camera control for video and single image acquisition used the ThorCam software application (Thorlabs). The color camera contains a 2448 × 2048 pixel CMOS sensor, with 3.45 µm × 3.45 µm pixels, with the integral IR-blocking filter replaced with a 280 nm long pass filter (FSR-WG280; Newport Corp, Irvine, CA, USA). Removal of the blocking filter is required for NIR sensitivity. The camera maximum frame rate is 53 frames per second, with the actual frame rates governed by the exposure times required for different illumination wavelengths and intensities (typically 5 ms to 160 ms per image). A 100 W tungsten-halogen microscope lamp (Olympus model U-LH100) was the source for broad-spectrum light, and the means to select different bands of light was either a Sutter Lambda 10-3 10-position filter wheel (Sutter Instruments, Novato, CA, USA) placed between the microscope lamp and the microscope illumination port, or a Thorlabs CFS1 filter slider placed between the microscope camera port and color camera.

Table 1 lists the different filters used for illuminating the various stains in work presented here. The filters are listed with each specimen, biomarker, and chromogen they are used with and the figures in which images of these specimens are displayed. For all assays, the H&E stain was viewed with the tungsten microscope lamp through a broadband visible filter (85% transmission 420–690 nm, 10HMR-0 hot mirror, Newport Corp.). When using the tungsten lamp, removal of the IR-blocking filter within the tungsten lamp housing is required to access the NIR light. Tungsten lamp emission versus wavelength with the IR-blocking filter removed is plotted in Supplementary Figure S6B, together with the filtered lamp emission for viewing and imaging H&E (visible white light) and each of the four invisible CDCs. These can be compared with stain absorbance spectra plotted in Figure 1B. Sufficient deep blue/UV light is available for CDCs absorbing near 400 nm (e.g., NMC). However, low-level light near 375 nm for AMC absorbance requires camera acquisition times on the order of one or more seconds per frame, so light-emitting diode (LED) illumination is preferred (see below and Table 1). Filters can also be placed below the microscope stage (commercial microscope manufactures typically supply attachments for this purpose) or in the emission filter positions of a fluorescence/reflectance attachment. Alternatively, selection of different light bands can be achieved using LEDs, often combined with filters to narrow the wavelength range. A 365 nm LED combined with a 375 nm bandpass filter was used for imaging the AMC chromogen. Multiple LEDs and broadband light sources were combined as described for the dual-camera system [5], and described in the Supplementary Methods. Since both the visible H&E stain and IHC stain(s) are viewed on the color camera alone, the dual-camera mount and associated beam splitters and filtering required for the dual-camera system are not required, considerably simplifying the system hardware. However, as a precaution used with the dual-camera system, filters were placed within each eyepiece to prevent light from the invisible illumination channels reaching the eye (custom filter ET 560/280, 24 mm diameter × 1.1 mm, unmounted, Chroma Technology, Bellows Falls, VT, USA). Greater detail of the microscope system hardware and different configurations are included in the Supplementary Methods.

### 2.5. Imaging and Image Processing

In addition to interactive viewing at the microscope, multispectral imaging with different filters and/or LEDs can be performed with either the monochrome camera (dual system) or color camera. Multispectral imaging with the monochrome camera has been described [8] and multispectral imaging with the color camera is essentially the same but uses color images converted to monochrome or, preferably, uses the color plane (red, green, or blue) of each color image matched best to each chromogen’s peak absorbance. The selected color planes are monochrome images that can be used in spectral unmixing to remove spectral crosstalk due to overlapping dye absorbance spectra. The unmixed images are then used to form composite images or perform other digital image analysis, in the same manner as performed for monochrome brightfield or fluorescence images [8,9,10,11]. Reference coefficients for the spectral unmixing of images recorded with the color camera were calculated in the same manner as with monochrome cameras [8]. This utilized tonsil FFPE specimens, each stained with a single dye without counterstain, using the same filter or LED and same color plane as in the imaging of the multiplexed specimen. Linear unmixing and composite image formation were performed using macros written for ImageJ (version 1.51j8) [12] and MATLAB (Mathworks, Natick, MA, USA, 2022a). Commercial (e.g., Halo, Indica Labs, Albuquerque, NM, USA) and publicly available (e.g., QuPath, https://qupath.github.io/ (accessed on 28 November 2024) and ImageJ) software packages have been used for visualization and quantitative evaluation of unmixed images [13]. ThorCam software (Thorlabs, Inc., Newtown, NJ, USA, ThorCam Version 3.2.1) was used for camera control and image acquisition. Absorbance spectra of chromogens and conventional dyes on FFPE tonsil tissue, plotted in Figure 1B, were recorded on light transmitted through the specimen slide using a spectrometer as described previously [5]. All microscope images presented here used the full camera sensor and the 20× microscope objective, providing a field of view of 422 µm × 353 µm.

## 3. Results

### 3.1. Microscope Systems

**Figure 2:**
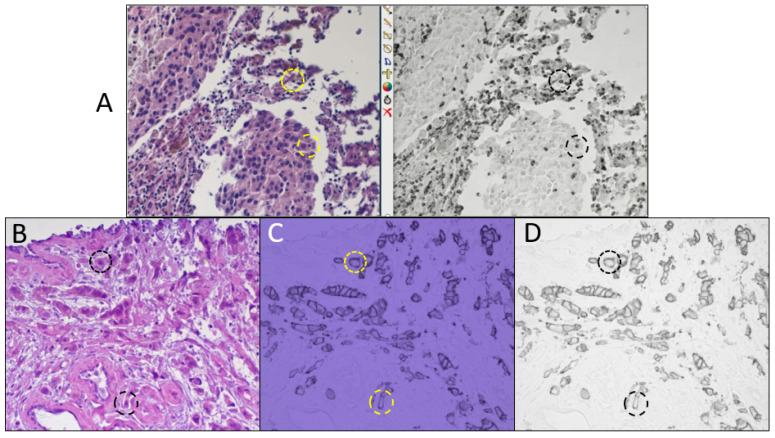
H&E + invisible IHC images using the dual-camera microscope system (**A**) and single-camera microscope system (**B**–**D**). (**A**) Dual-camera microscope monitor screenshot simultaneously displaying visible H&E staining (left side, color camera video) and non-visible LAG3 sCy7 IHC staining (right side, monochrome camera video) of melanoma FFPE tissue. The specimen was simultaneously illuminated with white light from a tungsten microscope lamp through a broad white light filter (420–620 nm) and NIR light from a 770 nm LED. (**B**,**C**) Sequential view of H&E staining (**B**) and non-visible HER2 IHC using ir870 chromogen (**C**), on breast tumor FFPE tissue, using same color camera (IR-blocking filter removed). The specimen was illuminated with a tungsten microscope lamp while switching between the broad white light filter (**B**) and an 880 nm bandpass filter (**C**). (**D**) HER2 image displayed in grayscale (blue color plane of color image in part **C**). Circles highlight two individual cells in images of each specimen. The color saturation of (**C**) was reduced to 60% to aid in viewing the printed blue image.

Figure 2A shows a picture of the computer monitor of the dual-camera microscope system during the presentation of live side-by-side images of the color camera (left) and monochrome camera (right). The specimen is melanoma FFPE tissue stained with H&E and the biomarker LAG3 detected with the sCy7 chromogen. The specimen is illuminated simultaneously with white light from a tungsten-halogen microscope lamp and 770 nm NIR light from an LED light source. The visible white light (showing the H&E staining pattern) is transmitted to the color camera and the invisible NIR light (showing LAG3 expression) is reflected to the monochrome camera. The simplified single color camera system can provide similar images but instead of two side-by-side simultaneous live images, a single live image is presented which can alternate between the visible H&E stain and the invisible chromogen stain by switching illumination wavelengths. An example of this is shown in Figure 2B,C of breast tumor FFPE tissue stained with H&E (Figure 2B) and HER2 IHC using the ir870 chromogen (Figure 2C). In Figure 2B,C, all light was supplied from a single tungsten microscope lamp, switching between a white light transmitting filter for H&E and an 880 nm single-bandpass filter for the NIR light absorbed by the ir870 chromogen. Note that the color camera image at 880 nm (Figure 2C) shows a bluish-hue. The visible presentation of the invisible chromogens depends upon the responsivity of each color pixel at the invisible wavelength (all three pixels are responsive at 880 nm with the integral camera IR-blocking filter removed) and the white balance setting of the color camera. The spectra of the red, green, and blue pixel responses for the camera used here are plotted in Supplementary Figure S6A. Since human color perception can vary between individuals, and deep blue can be difficult to discern, conversion to grayscale, as performed in Figure 2D, removes the color dependence to improve perception. The color saturation of the blue image in Figure 2C (and other figures, as noted in the legends) has been decreased to 60% since the printed blue images can be difficult to visually interpret, compared to direct viewing through the microscope or viewing images presented on a color monitor.

### 3.2. H&E Plus Multiplex IHC of Clinical Specimens

**Figure 3:**
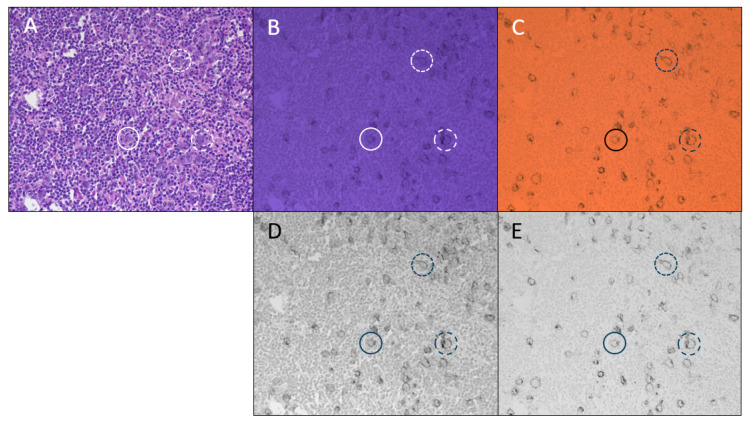
Hodgkin’s lymphoma FFPE specimen, 2-plex IHC (CD30 AMCA/CD15 sCy7) + H&E, imaged with color camera (IR-blocking filter removed). (**A**) H&E imaged under white light illumination (tungsten microscope lamp with IR-blocking filter removed plus 420–690 nm broadband filter). (**B**) CD30 IHC using AMC chromogen and illuminated with UV light (365 nm LED plus 375 nm single bandpass filter). (**C**) CD15 IHC using sCy7 chromogen with NIR illumination (tungsten lamp with IR-blocking filter removed plus 769 nm single bandpass filter). (**D**) Grayscale representation of color image B (CD30; blue image plane). (**E**) Grayscale representation of color image C (CD15; red image plane). Circles highlight three individual cells under different illumination. The color saturation of (**B**) was reduced to 60% to aid in viewing the printed blue image.

A duplex IHC plus H&E of a Hodgkin’s lymphoma FFPE specimen is shown in Figure 3 with images recorded using the color camera (IR-blocking filter removed) while switching between white light illumination for H&E (Figure 3A), UV illumination for the AMC chromogen used to identify CD30 expression (Figure 3B), and NIR illumination for the sCy7 chromogen used to identify CD15 expression (Figure 3C). Grayscale images of the CD30 (Figure 3D) and CD15 (Figure 3E) are included. The membrane staining patterns of the CD30 and CD15 proteins are clearly discerned, although a small amount of nuclear staining is visible in the CD30 (AMC) image. This is due to the broad hematoxylin absorbance that extends in a small degree to the UV where AMC absorbs light (see hematoxylin and AMC absorbance spectra in Figure 1B).

**Figure 4:**
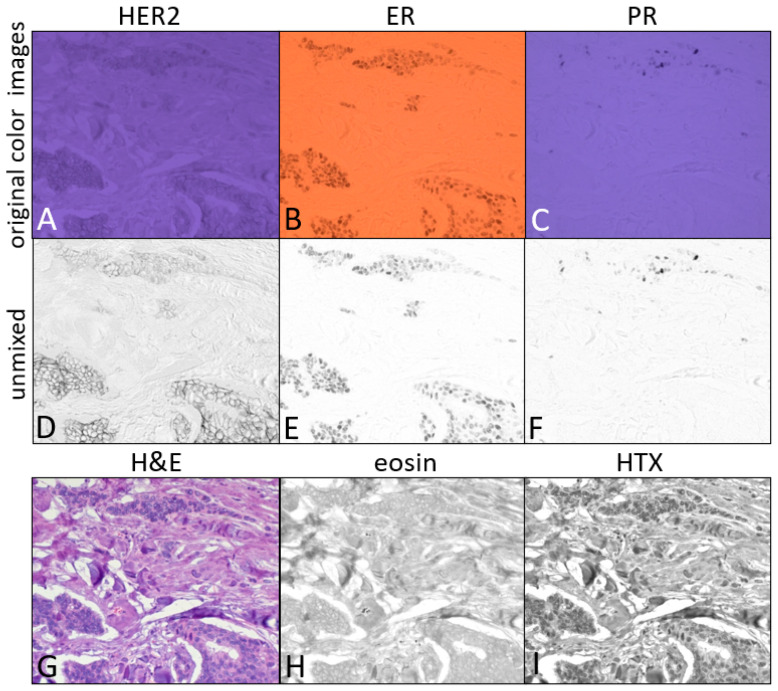
Breast tumor 3-plex IHC (HER2, ER, PR) + H&E recorded using the color camera (IR-blocking filter removed). Images were recorded with 375 nm light for HER2 (**A**), 769 nm light for ER (**B**), 880 nm light for PR (**C**), and white light for H&E (**G**). All light channels were acquired with filtered tungsten light except for the 375 nm LED channel. Spectral unmixing of the 5 images produced the unmixed HER2 (**D**), ER (**E**), PR (**F**), eosin (**H**), and hematoxylin (**I**) images. The color saturation of (**A**,**C**) was reduced to 60% to aid in viewing the printed blue images.

Viewing triplex IHC in the presence of H&E with the color camera is shown in Figure 4 for breast tumor FFPE tissue stained with H&E (Figure 4G) and IHC for HER2 (Figure 4A), estrogen receptor (ER; Figure 4B), and progesterone receptor (PR; Figure 4C). In addition to the UV-absorbing AMC chromogen staining HER2, two NIR-absorbing chromogens are used; sCy7 to stain ER, and ir870 to stain PR. Three filters in the tungsten lamp filter wheel were used to sequentially illuminate H&E, ER, and PR, and the 365 nm LED was used to illuminate HER2. Some of the dark-staining PR expressing cells can be seen in the ER color image due to ir870 absorbance in the 769 nm light channel (see ir870 and sCy7 absorbance spectra in Figure 1B), referred to as spectral crosstalk or bleed-through. As with monochrome cameras typically used in multispectral imaging, spectral unmixing can be applied to these color camera images to remove crosstalk. Spectral unmixing utilized the 365 nm (HER2), 769 nm (sCy7), and 880 nm (ir870) color images, as well as images recorded at 520 nm for eosin and 599 nm for hematoxylin, producing the unmixed images of HER2 (Figure 4D), ER (Figure 4E), PR (Figure 4F), eosin (Figure 4H), and hematoxylin (Figure 4I).

**Figure 5:**
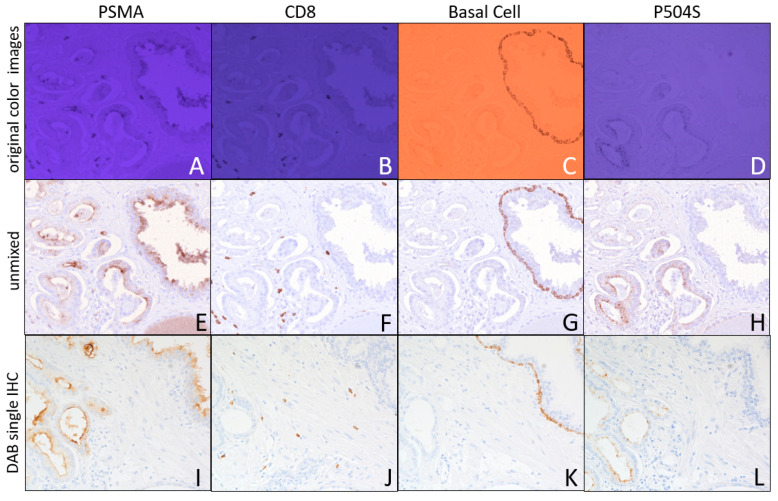
Multispectral imaging with color camera of H&E plus 4-plex invisible IHC on prostate FFPE tissue illuminated with 6 light channels (five filtered tungsten light and 375 nm LED) to identify PSMA, CD8, basal cell, and P504S expression. (**A**–**D**) Color images of PSMA, CD8, basal cell, and P504S staining, respectively. (**E**–**H**) Spectrally unmixed images of PSMA, CD8, basal cell, and P504S expression, respectively, combined with the unmixed hematoxylin image, applying brown pseudo-color to biomarker and blue pseudo-color to hematoxylin. Spectral unmixing used the 4 biomarker images (**A**–**D**) plus eosin (510 nm) and hemoxylin (599 nm) images. (**I**–**L**) Single DAB IHC plus hematoxylin counterstain for each of the four biomarkers, PSMA, CD8, basal cell, and P504S, respectively, on serial sections. The color saturation of (**B**,**D**) was reduced to 60% to aid in viewing the printed blue images.

**Figure 6:**
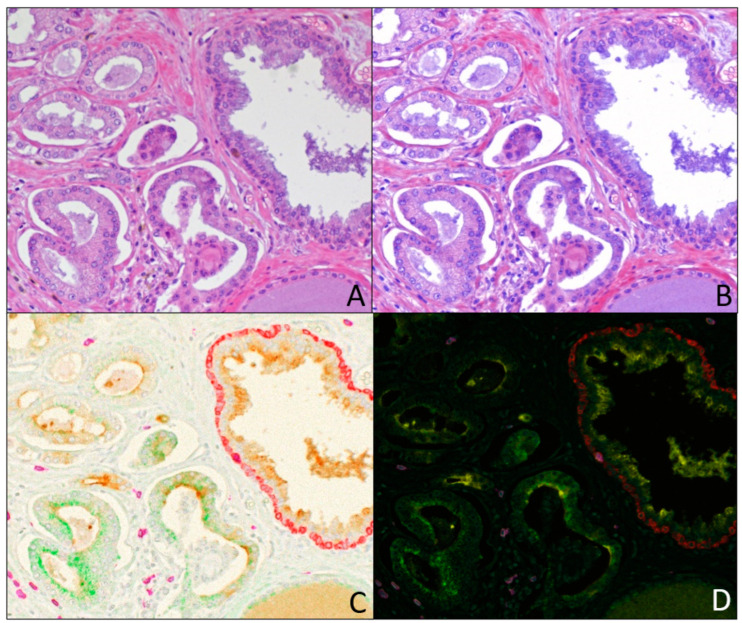
Composite images from 4-plex IHC + H&E on prostate tumor FFPE tissue. H&E image recorded under white light illumination (**A**) is compared to composite H&E image (**B**) prepared from unmixed eosin (recorded at 530 nm) and hematoxylin (recorded at 599 nm) images. (**C**) Composite image of all 4 chromogens plus hematoxylin to produce 5-color brightfield (transmittance) representation. (**D**) Composite image of all 4 chromogens plus hematoxylin to produce 5-color fluorescence-like (absorbance) representations. Unmixed biomarker images are presented in Figure 5.

Quadplex IHC that interferes minimally with H&E interpretation was demonstrated using two UV-absorbing chromogens and two NIR-absorbing chromogens. Figure 5 presents images of AMC and NMC UV-absorbing chromogens used to identify the PSMA and CD8 expression, respectively, and sCy7- and ir870 NIR-absorbing chromogens to identify the basal cell marker (cytokeratin 34 beta E12 plus p63) and P504S expression, respectively, in prostate tumor FFPE tissue. Figure 5A through Figure 5D show the color camera images of AMC, NMC, sCy7, and ir870 chromogens, respectively, and shows noticeable crosstalk between the AMC (365 nm) and NMC (405 nm) channels, in addition to the bleed-through from the ir870 (880 nm) channel into the sCy7 (769 nm) channel. The H&E image recorded under white light illumination is shown in Figure 6A. While the different markers are distinguishable from one another based on staining morphology, spectral unmixing becomes necessary for detailed interpretation. Spectral unmixing of the color camera images is shown in Figure 5E though Figure 5H for the PSMA, CD8, basal cell, and P504S, respectively. Included in the unmixing were the hematoxylin and eosin light channels (six light channels in total). Serial sections stained with a single DAB IHC plus hematoxylin counterstain are presented in Figure 5I through Figure 5L, respectively, for PSMA, CD8, basal cell, and P504S expression, showing a correspondence to the multiplex IHC.

The unmixed biomarker images in Figure 5E through Figure 5H are actually composite images of the respective unmixed biomarker images plus the unmixed hematoxylin image, with pseudo-coloring imitating DAB IHC (brown) with hematoxylin counterstain (blue). The H&E stain was also re-constructed as a composite image of the unmixed hematoxylin and eosin images, shown in Figure 6B (compared to the Figure 6A color image recorded under white light). More complex composite images were generated, including five-color composite images of the four protein markers plus hematoxylin in brightfield (Figure 6C) and fluorescence-like (Figure 6D) representations.

### 3.3. DAB IHC Plus Invisible IHC

**Figure 7:**
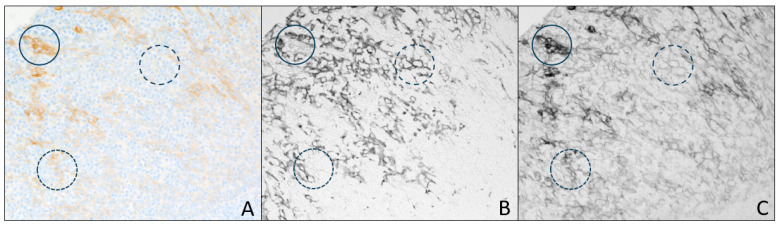
Breast cancer FFPE tissue stained with HER2 DAB and PD-L1 sCy7 duplex IHC, plus hematoxylin counterstain, viewed with white light from tungsten lamp, (**A**) showing DAB and hematoxylin counterstain (color camera image), 769 nm filtered tungsten light, (**B**) showing sCy7 staining (monochrome camera image), and 405 nm light, (**C**) showing the DAB absorbance (monochrome camera image). Circles highlight several cell groups to compare PD-L1 and HER2 expression patterns.

The application of invisible IHC to augment visible IHC is shown in Figure 7 in which a breast cancer FFPE tissue was stained with HER2 IHC plus hematoxylin counterstain using the common brightfield chromogen, DAB, as well as PD-L1 IHC using the sCy7 chromogen. Figure 7A displays the color image with white light illumination, showing the HER2 expression and nuclear hematoxylin staining. Figure 7B shows the PD-L1 sCy7 staining under NIR illumination. These images were recorded using the dual-camera system so the color video of H&E and monochrome video of sCy7 were viewed simultaneously on the computer monitor. Figure 7C was imaged with 405 nm light, which emphasizes the DAB absorbance over hematoxylin and sCy7 to isolate the HER2 stain. Circles around several cell groups highlight the difference in PD-L1- and HER2-staining patterns.

## 4. Discussion

### 4.1. Microscope Systems and Interactive Specimen Evaluation

Performing H&E staining and IHC on the same tissue section, and viewing simultaneously or sequentially, provides several important benefits, including the conservation of small specimens and the ability to combine protein expression with the extensive cellular and tissue organizational information H&E reveals. Identifying which cells are positive for various biomarkers, and combinations of biomarkers, in the case of multiplex IHC, together with their definitive location relative to each other and their locations relative to the morphological landscape, provides valuable information that can be tested for the association with disease diagnosis, prognosis, and therapeutic response [14,15,16]. Additionally, having the morphological and protein expression jointly available could increase the pathologist’s confidence in difficult diagnoses and possibly reduce the turnaround time. The ability to combine up to four biomarkers with H&E on the same slide, demonstrated here, provides fertile ground for the application of machine learning and artificial intelligence, and further reduces the need to use multiple serial sections to analyze the biomarker expression in cancer patients. We have developed a new approach that applies non-visible deposited chromogens to the IHC, allowing the simultaneous use of both H&E and IHC [5] such that the conventional H&E staining can be evaluated with minimal interference from deposited chromogen absorbance, and IHC stains can be evaluated with minimal interference from H&E absorbance.

By using CDCs that absorb light outside of the visible spectrum, a camera system sensitive to UV through NIR light, and illumination supporting that range are necessary. We previously described a two-camera microscope system that simultaneously presents live images of the visible H&E staining (color camera) alongside video of the invisible chromogenic stain (monochrome camera) on the computer monitor [5]. This system allows the pathologist to sit at the microscope and visually scan the specimen while manually translating the stage, in the same manner that clinical specimens are typically evaluated. The familiar view of H&E through the microscope’s eyepieces is also available. The depiction of a computer monitor displaying both H&E (color video) and LAG3 IHC (NIR monochrome video) of a melanoma FFPE specimen presented in Figure 2A demonstrates how the same individual cells can be located within both images, providing their LAG3 expression status and H&E staining of surrounding tissue (example cells indicated with circles). The system can also view multiplex IHC with multiple invisible CDCs by switching between different UV and/or NIR illumination channels.

The system simplification we report here using a single color camera reduces the system cost (one camera and fewer hardware/optical components) and looks very similar to the standard brightfield microscope system commonly used by pathologists, which includes the microscope, tungsten-halide lamp, and a color camera (Figure 1A). The difference is the inclusion of a means to select illumination wavelengths, such as a filter slider, filter wheel, and/or LEDs. If the microscope already includes holders for filters, either in the base of the microscope or in a reflectance/fluorescence module, then the only additional costs, in the simplest version, would be for optical filters. These include bandpass filters to select the desired invisible chromogen absorbance bands, a broadband white light filter for viewing the conventional stains (several hundred dollars each), and a filter for each eyepiece that blocks UV and IR light from exiting the eyepiece for the viewer’s safety. If the microscope does not already have a filter holder, then these can be supplied by microscope or filter wheel manufactures, or an inexpensive filter slider can be constructed as described in the Supplementary Methods. Although we have included tungsten lamps as illumination sources, these can be replaced with LEDs to provide the visible white light in addition to individual dye-specific wavelengths. Also necessary are the removal of a filter in the lamp housing, that blocks near IR and some UV transmission, and a filter in the color camera that otherwise restricts the camera to visible light (not all color cameras provide this option). The primary disadvantage of the current single-camera system is that the H&E and IHC videos cannot be viewed simultaneously but must be sequentially viewed by switching between broad-spectrum white light and one or more invisible light channels, for example, as shown in Figure 2B (white light illumination) and 2C (NIR illumination) to alternately display the H&E and HER2 IHC. Once more, there is a one-to-one correspondence between the same cells in the two images (example cells indicated by circles). The visual appearance of the color camera images of the invisible CDCs is determined by the responsivity of the red, green, and blue pixels at the illumination wavelengths, and the camera white balance. To avoid differences in visual acuity with different colors, color images can be converted to grayscale (Figure 2D). All of the images in Figure 3, Figure 4 and Figure 5 are recorded using the single color camera system to demonstrate the utility of the simpler configuration.

### 4.2. Applications of Combined H&E and Invisible IHC to Clinical Specimens

The identification of Reed/Sternberg cells is important in the diagnosis of Hodgkin’s lymphoma. These are typically large multinucleated cells [17] identifiable by H&E staining, although they often comprise a small fraction of total cells and may be confused with other large atypical cells [18]. For this reason, IHC is an important diagnostic tool for Hodgkin’s lymphoma diagnosis since Reed/Sternberg cells commonly express CD30 and CD15, in the majority of cases [19]. Figure 3 shows the combination of H&E with dual CD30 and CD15 IHC to bring H&E and both biomarkers into the diagnosis of Hodgkin’s lymphoma. Figure 3A shows the H&E in which a number of large cells are visible and Figure 3B and Figure 3C show CD30 and CD15 staining, respectively (grayscale renditions pictured below each). The coincidence of the CD30 and CD15 expression clearly supports the assignment as Reed/Sternberg cells (several example cells are indicated by circles).

Strong predictors of a therapeutic response in breast cancer are the estrogen receptor and progesterone receptor status and HER2 amplification, leading to the selection of anti-HER2 [20] versus endocrine therapies [21]. Monitoring all three on a single specimen slide is demonstrated in Figure 4 together with H&E to guide the biomarker evaluation of the tumor regions. The breast tumor depicted here shows the less common expression of all three biomarkers, with HER2 viewed with UV illumination (Figure 4A), and ER and PR viewed under two different NIR wavelengths (Figure 4B and Figure 4C, respectively). These color images show that the longer wavelength NIR channel (880 nm) bleeds, to some extent, into the lower-wavelength NIR channel (769 nm) as seen in the several dark-staining PR-expressing cells in the upper right of the two images. Spectral unmixing removed the bleed-through, as can be seen comparing Figure 4E,F. Not only are all three biomarkers expressed in this specimen, but the co-expression of HER2 and ER is found in nearly all HER2-positive cells, clearly identified by switching between UV and NIR illumination to see the expression of both in the same cells. The co-expression of all three biomarkers is evident in some of the more weakly HER2-expressing cells in the upper portion of the microscope field.

In prostate cancer, the basal cell cocktail, P504S, and PSMA are important biomarkers [22,23] with high-molecular-weight cytokeratin and p63 marking basal cells in benign regions and an elevated P504S and PSMA expression often associated with cancer. Multiplex IHC for these three markers plus ERG and Ki-67 using four chromogens (Ki-67 and PSMA sharing one chromogen) has been demonstrated [23], and, here, we combine H&E with four invisible chromogens to identify the basal cell, PSMA, and P504S with a CD8 expression, for additional information on cytotoxic T cells. This was carried out by including two UV-absorbing chromogens, AMC and NMC, and two NIR-absorbing chromogens, sCy7 and ir870. An examination of Figure 5A through Figure 5D shows noticeable spectral crosstalk between the AMC and NMC UV channels, and from the ir870 NIR channel into the sCy7 NIR channel. If the expression of the different markers is confined to distinct cellular compartments or different cells types, the level of bleed-through may still permit a visual analysis while sitting at the microscope. However, while the pathologist may be able to distinguish between the PSMA and CD8 expressing cells, and the P504S and basal cell marker expressing cells from each other based on characteristic staining patterns, the crosstalk can be removed by image processing to spectrally unmix the four biomarker stains from each other and the eosin, and hematoxylin staining. The unmixed biomarkers, presented in Figure 5E through Figure 5H, each include the unmixed hematoxylin image, with pseudo-coloring to mimic the individual DAB biomarker IHC (brown) plus hematoxylin counterstain (blue). The unmixing also clarifies any potential co-expression of biomarkers. Compared to DAB IHC for the four markers individually in the same field on serial sections (Figure 5I through Figure 5L), the 4-plex IHC faithfully represents the four markers while providing all biomarker expressions on the exact same set of cells with no changes in morphology due to sectioning through the tumor, thereby providing exact cell-to-cell relationships, all within the H&E context (Figure 6A).

Counting the hematoxylin and eosin information, the H&E plus 4-plex IHC staining of the prostate specimen constitutes a six-color example of brightfield multispectral imaging. While a monochrome camera is typically used with multispectral imaging, this example shows that a color camera can replace the monochrome camera. Image processing of the multispectral color camera images, after spectral unmixing, provides many choices for combining the unmixed images into composite images to aid visual evaluation. This ranges from the simple combination of the individual biomarker and hematoxylin images (Figure 5E through Figure 5H) to mimic the familiar DAB IHC plus counterstain, to the combination of multiple biomarkers to investigate the relationships between different cell populations, identified by different biomarker expression patterns. Figure 6B shows an H&E composite image constructed from the unmixed hematoxylin and eosin channels, which compares well with the H&E color camera image recorded with white light illumination (Figure 6A) and allows us to adjust the relative staining intensity of the two dyes to fit personal preference. The inclusion of hematoxylin and/or eosin in the composites adds the foundational interpretive value afforded by H&E staining to the relationships between cellular populations. The combination of all four unmixed biomarkers with unmixed hematoxylin is shown in both brightfield (Figure 6C) and fluorescence-like representations (Figure 6D) to show all cellular relationships. Composite images can simplify visualization by showing only two or three biomarkers at a time in the same composite image. Unmixed images are amenable to further processing by software designed for the image analysis of pathology specimens (e.g., Halo (Indica Labs, Albuquerque, NM, USA, HALO AI 3.5.3577), Visiopharm (Hoersholm DNK, ), or QUpath, Belfast, UK, Version: 0.5.0) [13].

### 4.3. DAB IHC + Invisible IHC

DAB is the most common chromogen used clinically in IHC, and, therefore, pathologists have the most familiarity interpreting this stain. Observing the fine details of staining is particularly important for HER2, since therapy selections depend upon distinguishing HER2 2+ and 3+ from a lower expression [24], and, with the advent of anti-HER2 drug conjugates, identifying HER2 1+ versus 0 is of significant interest [25,26]. DAB’s broad absorbance spectrum across visible wavelengths can complicate multiplexing with conventional visible absorbing deposited chromogens, but the use of invisible CDCs reduces the absorbance interference between deposited chromogens, thereby permitting the use of the popular DAB chromogen in multiplexed IHC.

In the combined use of HER2 DAB IHC with PD-L1 sCy7 IHC, shown in Figure 7 using the dual-camera microscope system, the exact location of the PD-L1-expressing cells relative to the HER2-overexpressing cells is apparent. Since both markers are expressed by tumor cells in the membrane compartment, the use of the invisible deposited chromogen to stain PD-L1 and DAB to stain HER2 helps distinguish the two biomarkers, even in the same compartment of co-expressing cells, whereas the use of two visible-light-absorbing deposited chromogens would obscure each other’s detection in cells with co-expression. Switching between the NIR illumination of sCy7 and 405 nm light, highlighting DAB, provides further confidence in determining which cells are expressing combinations of the two biomarkers. Circles added to Figure 7 highlight several cell groupings which indicate, in this tumor section, that cells expressing high levels of PD-L1 have relatively low levels of HER expression, and vice versa.

### 4.4. Assay Considerations

When designing assays combining H&E with IHC stains, various parameters must be considered. One is the type of chromogen, and the CDCs [5,6], as used in this work, are important components, as a covalent attachment of each chromogen molecule to cellular and tissue components ensures that the IHC stains will be unaffected by the subsequent hematoxylin and eosin staining steps, or DAB IHC. In the workflow, performing the H&E staining first is not possible since the eosin is easily removed by the IHC processing and hematoxylin is significantly diminished. In addition to performing the IHC first, the order of biomarker staining in multiplex IHC is important since different primary antibodies are more sensitive to prior processing steps, such as heating to remove preceding antibodies (if required), while other primary antibodies may require the additional prior “conditioning” steps to expose the antigen. Additionally, some CDCs, including the cyanine dyes (Cy7, ir870) are sensitive to the H_2_O_2_ oxidation steps used when depositing the tyramide-modified CDCs, and are best placed later in the multiplex protocol.

Performing the H&E last in the protocol prevents the IHC steps from removing the hematoxylin or eosin; however, the IHC processing steps can affect the uptake of the hematoxylin and/or eosin stains. For example, we found hematoxylin staining to be noticeably darker following IHC in FFPE prostate tumor tissue, and, to compensate, we reduced the hematoxylin staining time from 2 min to 10 s to obtain a desired result. Thus, for a particular marker panel and protocol, optimization may be necessary to achieve the preferred depth of hematoxylin and eosin staining. Importantly, two groups have reported that prior IHC still resulted in faithful H&E and did not interfere with the IHC interpretation [3,4]. Excess hematoxylin staining, however, can create noticeable bleed-through to the UV channels, while having little effect on the NIR channels. Heavy staining by any chromogen can potentially lead to the non-specific adsorption of dyes, and should be avoided.

Our single- and dual-camera systems enable interactive slide evaluation by the pathologist seated at the microscope, with the use of invisible CDCs permitting the evaluation of both H&E and IHC on a single stained tissue section. Keeping the number of invisible CDCs to one (UV or NIR) or two (one UV and one NIR) allows the pathologist to clearly view each chromogen while manually scanning the specimen, without the need for image processing to separate and reintegrate signals. Adding a third or fourth chromogen necessitates the use of two UV and/or two NIR CDCs, leading to some bleed-through of signals. If the different chromogens are staining biomarkers with distinctly different staining patterns, then crosstalk may be acceptable in many cases, but digital processing is available when staining patterns are too closely aligned.

## 5. Conclusions

In summary, we have demonstrated the ability to perform simultaneous H&E and IHC, making use of invisible deposited chromogens, to preserve the specimen and elucidate the relationships between the cell populations and cellular and tissue architecture. Two different microscope systems were described for viewing and imaging these simultaneously stained tissue sections, enabling pathologists to view these stains while seated at the microscope and manually scanning the specimen for relevant areas of interpretation. The dual-camera system permits viewing simultaneous live images of H&E and IHC while the simpler single-camera system permits the sequential viewing of the two videos. H&E staining can also be viewed through the eyepieces on both systems, and both systems support multi-spectral imaging. Examples of clinically relevant specimens, including melanoma, breast tumor, Hodgkin’s lymphoma, and prostate tumor, successfully demonstrated as a proof of principle H&E combined with single to 4-plex IHC. Invisible IHC was also combined with conventional DAB IHC to augment the familiar DAB stain interpretation, potentially adding additional biomarker information. As spectral crosstalk in 3- and 4-plex IHC can become an issue with some panels, multi-spectral imaging and spectral unmixing were used to remove crosstalk and provide a variety of composite images that aid the interpretation of complex expression relationships, with the potential for further evaluation using cell and tissue analysis software and AI-aided interpretation. While multispectral whole-slide imaging represents an attractive future state of the pathology laboratory, widespread adoption by pathologists and regulatory approval may be years away, so this technology presents a simpler, cheaper, and faster method to introduce multiplexed IHC into clinical practice. Therefore, the comparatively simple approach presented here to extend the multiplexing capabilities of anatomical pathology may fill the gap until whole-slide scanning is accepted and commonplace in the clinical laboratory.

## Figures and Tables

**Table 1:** H&E + IHC specimen and light channel information. Specimens were stained by single or multiplex IHC for the indicated biomarkers and chromogens. The illumination channel for each chromogen is listed, and figure numbers in which images of each are presented. H&E and DAB plus hematoxylin stains were viewed and imaged using visible white light illumination from a tungsten microscope lamp through a 420–690 nm (>85% transmission) filter. DAB was additionally viewed under 405 nm illumination. Additional filter and LED information, including sources, is listed in Supplementary Table S1.

Specimen	Conventional Stain	Biomarker	Chromogen	Optical channel		Figure
				Lamp	Filter *	
Melanoma	H&E	LAG3	sCy7	770 nm LED	none	2A
Breast tumor	H&E	HER2	ir870	tungsten	880/40	2B–D
Hodgkin’s lymphoma	H&E	CD30	AMC	365 nm LED	375/28	3
		CD15	sCy7	tungsten	769/49	
Breast tumor	H&E	HER2	AMC	365 nm LED	375/28	4
		ER	sCy7	tungsten	769/49	
		PR	ir870	tungsten	880/40	
	DAB IHC + hematoxylin	HER2	DAB	tungsten	405/30	7
		PD-L1	sCy7	tungsten	769/49	
Prostate tumor	H&E	PSMA	AMC	365 nm LED	375/28	5, 6
		CD8	NMC	tungsten	405/30	
		basal cell	sCy7	tungsten	769/49	
		P504S	ir870	tungsten	880/40	

* Filter parameters listed as center wavelength/FWHM, both in nm. FWHM = full width at half maximum transmission.

## Data Availability

Data generated as part of this study but not presented in the manuscript or supplementary material may be available from the corresponding author upon reasonable request and internal review.

## Supplementary Materials

The following supporting information can be downloaded at: https://www.mdpi.com/article/10.3390/diagnostics15020164/s1, Figure S1: Single-camera and dual-camera microscope configurations; Figure S2: Dual-camera microscope; Figure S3: Back of microscope displaying illumination system; Figure S4: Single-camera microscope with color camera at camera port (no filter changer at camera port); Figure S5: Single-camera microscope with color camera and filter slider at camera port; Figure S6: Spectral responses of multiple components in the imaging system; Table S1: Illumination channel lamps and filters.
