# Supplementary material for: Concurrent Viewing of H&E and Multiplex Immunohistochemistry in Clinical Specimens
Source: Diagnostics (Basel). 2025 Jan 13;15(2):164. doi: 10.3390/diagnostics15020164 (PMC11764411; doi:10.3390/diagnostics15020164)
Supplement: Supplementary file 1 [file diagnostics-15-00164-s001.zip › diagnostics-3368503-supplementary.pdf]

Supplementary Methods for Concurrent Viewing of H&E and Multiplex Immunohistochemistry in Clinical Specimens

IHC and conventional staining

Fully automated single and multiplex IHC using covalently deposited chromogens (CDCs) were performed on a DISCOVERY Ultra system (Ventana Medical Systems Inc. (VSMI); Roche Diagnostics Corp, Tucson, USA). In general, IHC was performed at 37°C, except as noted, and Reaction Buffer wash solutions were diluted from 10x concentrate (cat. no. 950-300). A slide-mounted paraffin section was de-paraffinized by warming the slide to 70°C for 3 cycles, each 8 min long. Antigen retrieval was performed by applying Cell Conditioning 1 (VMSI Cat. no. 950-124) and warming the slide to 94°C for 64 min. Staining of each biomarker was performed sequentially. Steps in each staining reaction included adding 0.1 ml primary antibody targeting that biomarker and incubating for 16-32 min, washing in Reaction Buffer to remove unbound antibody, adding 0.1 ml anti-species antibody targeting the primary antibody (either anti-mouse or anti-rabbit) conjugated to hapten (hydroxyquinoline; HQ) and incubating for 8 min, followed by addition of 0.1 ml anti-HQ HRP for 8 min, followed by another wash with Reaction Buffer. 0.1 ml of tyramide modified 7-amino-4-methylcoumarin-3-acetate (tyramide-AMC) or tyramide-modified dibenzocyclooctyne (tyramide-DBCO) was added and incubated for 4 min, followed by addition of 0.1 ml of 0.01% H_2O_2 in borate buffer, pH 8.5 and incubating for 8-48 min. Chromogen or DBCO deposition was followed by washing in Reaction Buffer, and when DBCO chemistry was used, adding 0.1 ml azide-modified chromogen, and incubating for 8-48 min, with a final wash in Reaction Buffer. If multiplex IHC, the slide was incubated with Cell Conditioning 2 (VMSI Cat# 950-123) at 100°C for 8 min, followed by washing in Reaction Buffer, and continuing with the next biomarker staining steps. At the conclusion of the staining run, the slides were washed with Reaction Buffer and/or a dilute detergent solution (0.2 g Dawn dish liquid (Proctor & Gamble, Cincinnati, OH) in 250 ml water) and rinsed with water. H&E staining was performed manually after IHC by incubating slides in Ventana HE 600 Hematoxylin solution (cat no. 07024282001) for 2 min, water for 2 min, Ventana H&E 600 Differentiating solution (cat no. 06544339001) for 1 min, water for 1 min, Ventana H&E 600 Bluing solution (cat no. 06544347001) for 1 min, water for 1 min, 95% ethanol for 30 s, Ventana HE 600 Eosin solution (cat no. 06544304001) for 1 min, 70% ethanol for 1 min, twice in 100% ethanol for 1 min each, and 3 times in xylene for 1 min each. Slides were then allowed to dry briefly and mounted with Richard Allan Scientific Cytoseal XYL (ThermoFisher Scientific), covering with a type 1.5 glass coverslip, or mounted on a Sakura Finetek USA (Torrance, CA) Tissue-Tek Glass Automated Coverslipper using type 1.5 coverslips. Incubation times in hematoxylin and/or eosin were varied to account for reduced or increased staining of tissues after IHC, for example hematoxylin staining was reduced to 10 s and eosin increased to 2 min for some prostate and breast specimens.

Primary antibodies and enzyme-antibody conjugates were used at the concentrations, volumes, and incubation times recommended by the manufacturer. Azide-modified CDC reagents and tyramide-DBCO, were added to slides in 100 µL volumes at concentrations ranging between 50 and 800 µM in VMSI Discovery TSA diluent (cat no. 000060900). Azide-modified chromogens were applied typically at the same concentration as used for the tyramide-DBCO. The concentrations of CDC solutions were typically 400 µM for tyramide-AMC, 600-800 µM for the azide of 7-(methylamino)coumarin-3-acetate (NMC), 50-300 µM for the azide of sulfo-Cyanine 7 (sCy7), and 50-200 µM for the azide of a novel chloro-Cy7 (ir870) CDCs. Chromogen solutions were optimized with various concentrations of DMSO in aqueous buffers for solubility to support automated staining.

<u>Microscope system for viewing and imaging H&E and invisible IHC</u>

Supplementary Figure S1 depicts microscope configurations used to view and image specimen slides stained with both H&E and invisible IHC. The base microscope was either a BX-51 or BX-63 microscope (Olympus, Waltham, NJ, USA) with Olympus UPLXAPO 20X (NA 0.80) and UPLXAPO 10X (NA 0.4) objectives. The dual-camera configuration, pictured in Supplementary Figure S2, used Kiralux 5.0 Mpixel cameras (Thorlabs, Inc., Newton, NJ, USA), one monochrome (model CS505MU) and one color (model CS505CU) coupled by a dual-camera mount (2SCM1-DC, Thorlabs). Both cameras use the same underlying 2448 x 2048 pixel CMOS sensor allowing alignment of the two camera images using the translational and rotational adjustments of the dual-camera mount. A custom dichroic beamsplitter (560 nm center wavelength, 280 FWHM, at 45° incidence, Chroma Technology, Bellows Falls, VT, USA) in the mount allows transmission of visible light to the color camera and reflects invisible light (below 420 nm and above 700 nm) to the monochrome camera. Illumination options include 100 W tungsten halogen microscope lamps and LED illuminators, either singly or combined using 50-50 neutral density or dichroic beamsplitters. The dichroic beamsplitter was identical spectrally to the beamsplitter used in the dual-camera mount. Supplementary Figure S3 shows two 100 W tungsten halogen lamp housings (Olympus U-LH100), one with the IR-blocking filter removed to provide invisible wavelengths, each with a Lambda 10-3 10-position filter wheel (Sutter Instruments, Novato, CA), and a liquid light guide from several LED illuminators, coupled together at the microscope lamp port. The light sources are couple via two pE Combiners (CoolLED, Andover, UK), the tungsten lamps being combined with a dichroic beamsplitter (same spectral characteristics as the dichroic beamsplitter in dual-camera mount), and the tungsten lamp outputs then combined with the LED liquid light guide using a 50-50 neutral density beamsplitter (Chroma Technology). More details of the dual-camera system may be found in Morrison LE, Lefever MR, Lewis HN, Kapadia MJ, Bauer DR. Conventional histological and cytological staining with simultaneous

immunohistochemistry enabled by invisible chromogens. Lab Invest. 2022;102(5):545-553.

The simpler single-camera design used only the color camera mounted at the camera port, as is typical for a brightfield microscope used by a pathologist. The color camera, however, must have the integral IR-blocking filter removed, which normally serves to restrict the camera image to visible wavelengths. This was achieved on the Thorlabs Kiralux 5.0 Mpixel CMOS color camera by removing the retaining ring at the camera opening. The blocking filter was replaced with an FSR-WG280 colored glass filter (Newport Corp, Irvine, CA, USA) with a cut-on wavelength of 280 nm and transmitting through the NIR. The response spectra of the red, green, and blue pixels, and the transmission spectrum of the IR-blocking filter in the Kiralux camera are plotted in Supplementary Figure S6A. The pixel response and integral IR-blocking filter transmission data for this camera were downloaded from the Thorlabs website (https://www.thorlabs.com/newgrouppage9.cfm?objectgroup_id=13255&pn=CS505CU#13262).

In the simplest single-camera configuration, the Kiralux camera was mounted at the microscope camera port using an Olympus C-Mount 0.63X Camera Adapter (U-TV0.63XC) and Thorlabs DC-CS1 C-mount-to-camera adapter. This is pictured in Supplementary Figure S4. In this configuration, selection of illumination channels was performed ahead of the camera port. The single-camera system may employ a single standard 100 W tungsten halogen microscope lamp (Olympus U-LH100) placed at the microscope illumination port. The lamp housing typically contains an integral IR-blocking filter which must be removed in order to utilize the NIR emission. The only other requirement is the placement of a filter holder/changer either at the camera port, the illumination port, or some other location between the two ports. Microscope manufacturers can supply filter holder/changers for this purpose or the emission filter holder can be used if the microscope is equipped with a fluorescence/reflectance attachment. Filters can also be manually placed in the light path below the microscope stage. As used here, a Sutter Lambda 10-3 10-position filter wheel was attached to the tungsten lamp housing at the microscope illumination port. With the tungsten lamp, camera acquisition times per frame ranged from 5 ms (769 nm filter) to 160 ms (405 nm filter) with 50% light split to the ocular. Filters used in this work are listed in Supplementary Table S1 with their center wavelengths, full widths at half maximum (FWHM), and suppliers. When using the AMC chromogen, tungsten lamp output in the UV (376 nm filter) required around 1 s acquisition time per frame, so a 365 nm LED (CoolLED pE-400 illuminator), with a 375 nm filter, was often used for imaging AMC (approximately 100 ms/frame). Tungsten lamp and LED (CoolLED pE-4000 16-channel LED illuminator) outputs were coupled using a pE Combiner (CoolLED) containing a 50-50 beamsplitter (Chroma Technology Corp.). For more flexibility, the number of selectable light channels were extended, as described for the dual-camera configuration above, by coupling two 100 W tungsten halogen lamp housings, each with a filter wheel and one with the IR-blocker removed for invisible channels, with the pE-4000 16 LED

illuminator (Supplementary Figure S3). Supplementary Figure S6B displays the unfiltered 100 W tungsten lamp emission spectrum, with the integral IR-blocking filter removed, and the lamp emission with a hot mirror (10HMR, Newport Corp.) in place to remove the NIR for normal viewing and color imaging of the H&E. Also plotted are the lamp's emission with the 880nm, 769 nm, 405 nm, and 375 nm filters in place. The insert in Supplementary Figure S6B depicts an expansion of the 380-430 nm wavelength region since the tungsten lamp output and transmission through the microscope optics is very low at the lower wavelengths. Emission was measured at the microscope stage using the microscope spectrometer described in reference 5 (Morrison, L.E.; Lefever, M.R.; Lewis, H.N.; Kapadia, M.J.; Bauer, D.R. Lab. Investig. 2022, 102, 545–553), employing an Ocean HDX UV to NIR spectrometer (Ocean Optics, Orlando, FL). No corrections were applied to the emission measurements.

As an alternative to placing the filters at the lamp port, a filter changer was placed at the camera port (see option in Figure 1A in main article) using the Thorlabs rod system, comprising a SM1A51 Olympus camera port adapter, CFS1 filter slider, LCP02 plate-to-rod adapter, four stainless steel rods of sufficient length to place the camera sensor at the microscope tube lens focal point, and an LCP4S rod to plate adapter. The LCP4S plate adapter was attached to the Kiralux color camera faceplate. This arrangement is pictured in Supplementary Figure S5. Note that for multispectral imaging, filter changers should be placed on the illumination side of the specimen to avoid potential image registration shifts with filter changes.

Note that for all microscope configurations, as a precaution, filters were placed within each eyepiece to prevent invisible light from the illumination bands from reaching the eye (custom filter, ET 560/280, 24 mm diameter x 1.1 mm, unmounted, ID number IN087569 to fit in reticle position, Chroma Technology). Alternatively, the oculars can be replaced with a tube lens without direct viewing capability (e.g. Olympus part U-TLU).

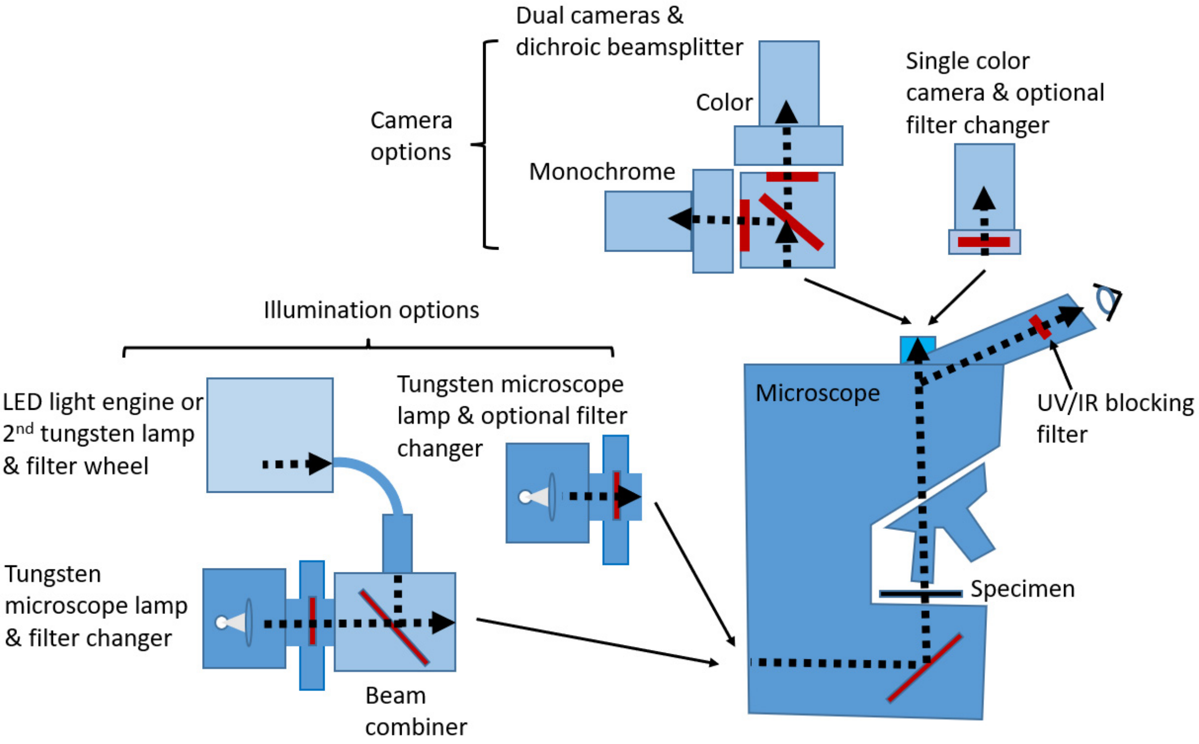

Supplementary Figure S1. Single-camera and dual-camera microscope configurations.

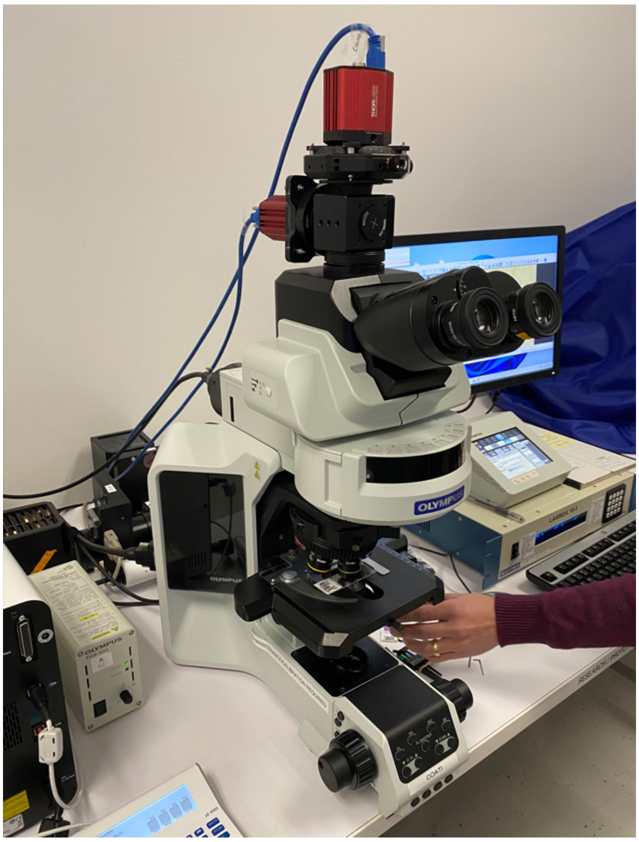

Supplementary Figure S2. Dual-camera microscope.

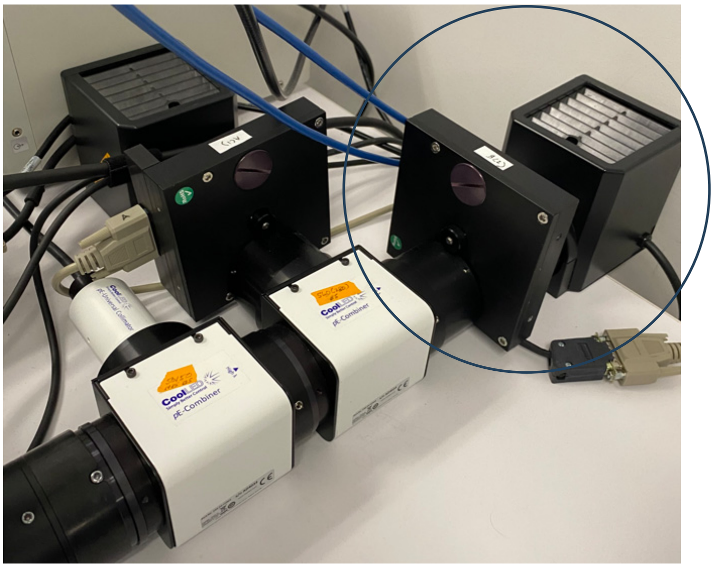

Supplementary Figure S3. Back of microscope displaying illumination system. Illumination provided by two 100 W tungsten halide lamps with filter wheels and LED illumination via light guide. Circle indicates tungsten lamp housing with filter wheel that

can be used alone at microscope lamp port in simple configuration. Filter wheel can be omitted if a filter changer is placed at the camera port (see below) or between camera and lamp ports.

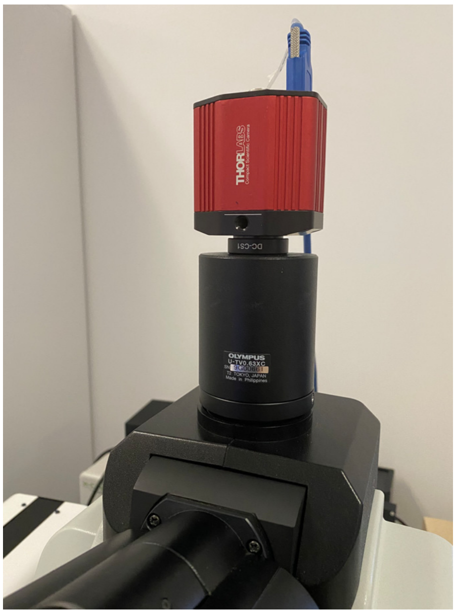

Supplementary Figure S4. Single-camera microscope with color camera at camera port (no filter changer at camera port).

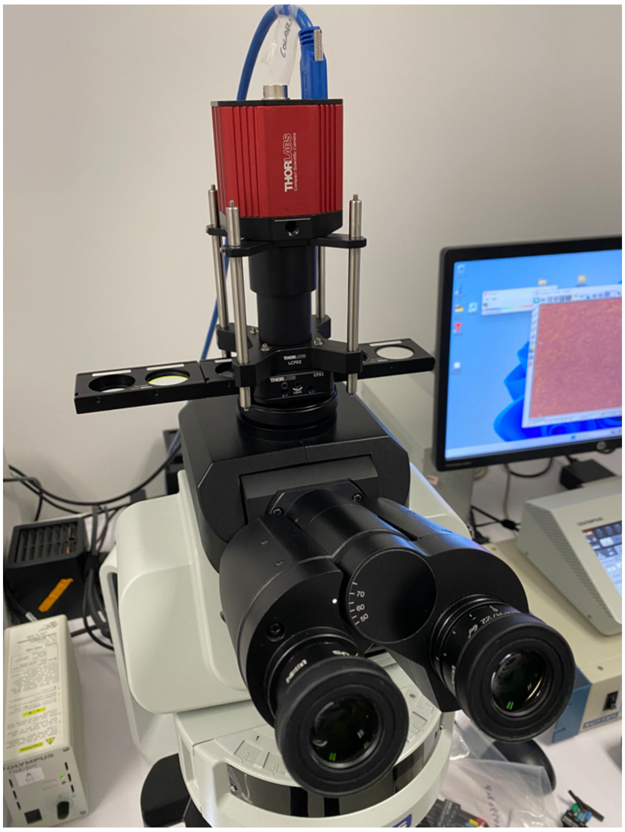

Supplementary Figure S5. Single-camera microscope with color camera and filter slider at camera port.

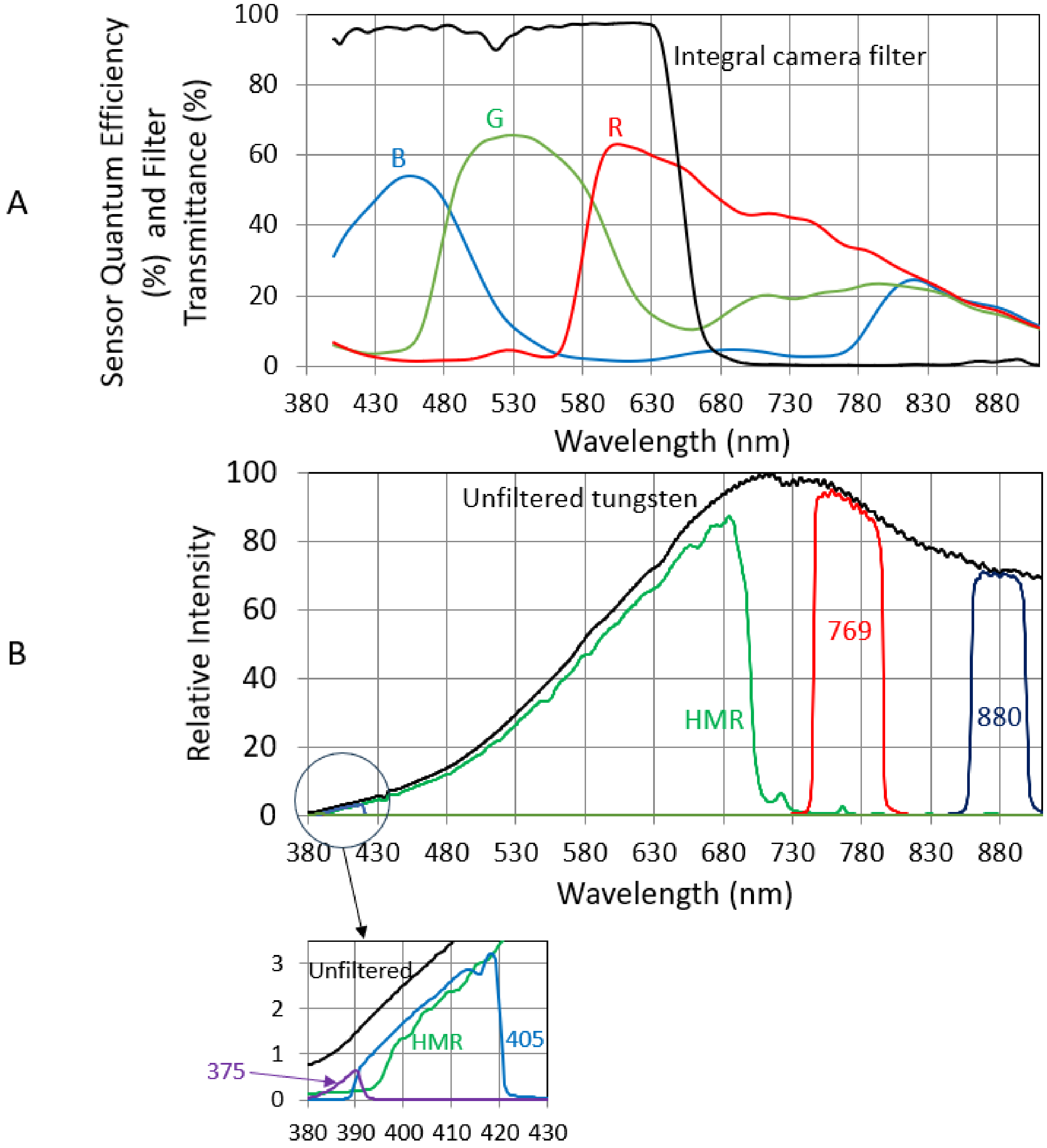

Supplementary Figure S6. Spectral responses of multiple components in the imaging system. A: Responsivity spectra of red (R), green (G), and blue (B) color camera pixels and transmission spectrum of integral camera IR-blocking filter in the Kiralux 5.0 Mpixel part number CS505CU color camera (Thorlabs, Inc.). The IR-blocking filter is removed in the single-camera microscope system for sensitivity to UV and NIR wavelengths. Pixel responsivity and filter transmission data are from the Thorlabs, Inc. website. B: Emission spectra of the tungsten microscope lamp with the integral IR-blocking filter removed, without and with additional filters as indicated in plots: HMR (10HMR-0 hot mirror) for imaging/viewing H&E stain, 880/40 nm bandpass filter for ir870 chromogen,

769/49 nm bandpass filter for sCy7 chromogen, 405/30 nm bandpass filter for NMC chromogen, and 375/28 nm bandpass filter for AMC chromogen (see Supplementary Table S1). Emission is expanded between 380 and 430 nm where lamp output and microscope transmission are low.

Illumination source	LED nominal λ, nm	bandpass filter, center λ/FWHM, nm	supplier	dye specificity
100 W tungsten halogen lamp			Olympus	
		10HMR-0 hot mirror, transmits 420-690nm; plus optional FGT165 temperature balancing filter	Newport Corp.; Thorlabs	H&E, DAB + HTX
		376/30 or 375/28	Semrock; Chroma Technology	AMC
		405/30	Chroma Technology	NMC
		510/15	Semrock	eosin
		599/13	Chroma Technology	hematoxylin
		769/49	Semrock	sCy7
		880/40	Chroma Technology	ir870
pE-4000 light engine			CoolLED	
	365	375/28	Chroma Technology	AMC
	405	unfiltered		NMC
	770	unfiltered		sCy7

Supplementary Table S1. Illumination channel lamps and filters.
